# Genomic patterns and characterizations of chromosomally-encoded *mcr*-*1* in *Escherichia coli* populations

**DOI:** 10.1186/s13099-020-00393-2

**Published:** 2020-11-28

**Authors:** Cong Shen, Lan-Lan Zhong, Furong Ma, Mohamed Abd El-Gawad El-Sayed Ahmed, Yohei Doi, Guili Zhang, Yang Liu, Songyin Huang, Hong-Yu Li, Liyan Zhang, Kang Liao, Yong Xia, Min Dai, Bin Yan, Guo-Bao Tian

**Affiliations:** 1 Department of Microbiology, Zhongshan School of Medicine, Sun Yat-sen University, 74 Zhongshan 2nd Road, Guangzhou, 510080 China; 2 Key Laboratory of Tropical Diseases Control (Sun Yat-sen University), Ministry of Education, Guangzhou, 510080 China; 3 Department of Clinical Laboratory Medicine, Third Affiliated Hospital of Guangzhou Medical University, Guangzhou, China; 4 Department of Microbiology and Immunology, Faculty of Pharmaceutical Sciences and Drug Manufacturing, Misr University for Science and Technology (MUST), Cairo, Egypt; 5 University of Pittsburgh School of Medicine, Pittsburgh, PA USA; 6 Departments of Microbiology and Infectious Diseases, Fujita Health University School of Medicine, Toyoake, Aichi Japan; 7 Department of Clinical Laboratory, Sun Yat-sen Memorial Hospital, Sun Yat-sen University, Guangzhou, China; 8 Department of Clinical Laboratory, Guangdong Provincial People’s Hospital/Guangdong Academy of Medical Sciences, Guangzhou, 510080 Guangdong China; 9 Department of Clinical Laboratory, The First Affiliated Hospital of Sun Yat-Sen University, Guangzhou, 510080 China; 10 School of Laboratory Medicine, Chengdu Medical College, Chengdu, 610500 China; 11 Department of Neonatal Surgery, Guangzhou Women and Children’s Medical Center, Guangzhou, China; 12 School of Medicine, Xizang Minzu University, Xianyang, 712082 Shaanxi China

**Keywords:** *mcr*-*1*, Colistin, Antimicrobial resistance, Genomic pattern, Chromosome, Insertion sequence, Phage

## Abstract

The emergence and transmission of the mobile colistin resistance gene (*mcr*-*1*) threatened the extensive use of polymyxin antimicrobials. Accumulated evidence showed that the banning of colistin additive in livestock feed efficiently reduce *mcr*-*1* prevalence, not only in animals but also in humans and environments. However, our previous study has revealed that a small proportion of *Escherichia coli* could continually carry chromosomally-encoded *mcr*-*1*. The chromosomally-encoded events, indicated the existence of stabilized heritage of *mcr*-*1* and revealed a potential threat in the antimicrobial stewardship interventions, are yet to be investigated. In this study, we systematically investigated the genetic basis of chromosomally-encoded *mcr*-*1* in prevalence and potential mechanisms of lineage, plasmid, insertion sequence, and phage. Our results demonstrated that the emergence of chromosomally-encoded *mcr*-*1* could originate from multiple mechanisms, but mainly derived through the recombination of IS*Apl1*/Tn*6330*. We reported a specific transmission mechanism, which is a phage-like region without lysogenic components, could associate with the emergence and stabilization of chromosomally-encoded *mcr*-*1*. These results highlighted the potential origin and risks of chromosomally-encoded *mcr*-*1*, which could be a heritable repository and thrive again when confronted with new selective pressures. To the best of our knowledge, this is the first study to systematically reveal the genomic basis of chromosomally-encoded *mcr*-*1*, and report a specific transmission pattern involved in phage-like region. Overall, we demonstrate the origin mechanisms and risks of chromosomally-encoded *mcr*-*1*. It highlights the need of public attention on chromosome-encoded *mcr*-*1* to prevent from its reemergence.

## Short report

The emergence and rapid dissemination of plasmid-mediated mobile colistin resistance gene (*mcr*-*1*) have become a severe threat to public health [1]. The predominant carriers of *mcr*-*1* were IncX4, IncI2, and IncHI2 plasmids, which are transferable and adaptive plasmid types with broad host range and contributed to the spread of *mcr*-*1* among various sources and bacterial species [2–4]. Besides, recombination of transposons, especially Tn*6330* (IS*Apl1*-*mcr*-*1*-*pap2*-IS*Apl1*), the primary vehicle for transmission of *mcr*-*1*, and phage-like sequences enable *mcr*-*1* to transfer across plasmids and isolates. Such contributed factors facilitated high *mcr*-*1* prevalence in several sources around the world, pushing local governments in Europe, Brazil and China to prohibit the use of colistin as growth promoter additive for livestock [5–8].

Accumulated evidence showed that banning of colistin in animal feed efficiently restricted *mcr*-*1* prevalence, not only in animals but also in humans and the whole ecosystem in China [2–4]. However, our previous study showed that a low proportion of *Escherichia coli* carrying chromosomally-encoded *mcr*-*1* continually existed in the ecosystem [4], which was sporadically reported by other studies as well [9–11]. On account of the plasmid that could be lost under certain circumstances due to instability, the chromosomally-encoded events could stabilize the heritage of *mcr*-*1*, threatening the intervention of colistin stewardship. In current study, we systematically investigate the epidemiological and genomic characterizations of *E. coli* population with chromosomally-encoded *mcr*-*1*.

Based on our previous large-scale epidemiological study from 2016 to 2018 in Guangzhou, China [4], we identified 24 (3.5%) out of 688 *mcr*-*1*-positive *E. coli* isolates with the chromosomally-encoded *mcr*-*1* (Table 1). The prevalence of chromosomally-encoded *mcr*-*1*-positive *E. coli* was from 0 to 9.8% for each source and from 2.2 to 4.8% for each epoch, indicating that the chromosomally-encoded *mcr*-*1* was at a low prevalence state in different dimensions (Table 1). Additionally, the comparison of prevalence for chromosomally-encoded *mcr*-*1* between different niches or epochs showed no significant difference (Fisher’s exact test, p > 0.05 for each comparison), suggesting that the emergence of chromosomally-encoded *mcr*-*1* was sporadic without temporal or source-specific signals.

**Table 1:** Prevalence of chromosomally-encoded *mcr*-*1* among 688 *mcr*-*1*-positive *E. coli* isolates

Sample source	Epoch (Oct 1 to Dec 31)			Total
	2016	2017	2018	
Pig	3.8% (3/78)	0% (0/63)	3.4% (2/58)	2.5% (5/199)
Healthy human carrier	9.8% (6/61)	4.0% (3/75)	0% (0/8)	6.3% (9/144)
Colonized patient	5.0% (3/60)	0% (0/41)	0% (0/9)	2.7% (3/110)
Infected patient	0% (0/27)	0% (0/17)	0% (0/11)	0% (0/55)
Food	7.4% (4/54)	3.9% (2/51)	0% (0/2)	5.6% (6/107)
Environment	0% (0/50)	4.5% (1/22)	0% (0/1)	1.4% (1/73)
Total	4.8% (16/330)	2.2% (6/269)	2.2% (2/89)	3.5% (24/688)

Data are % (n/N)

**Fig. 1:**
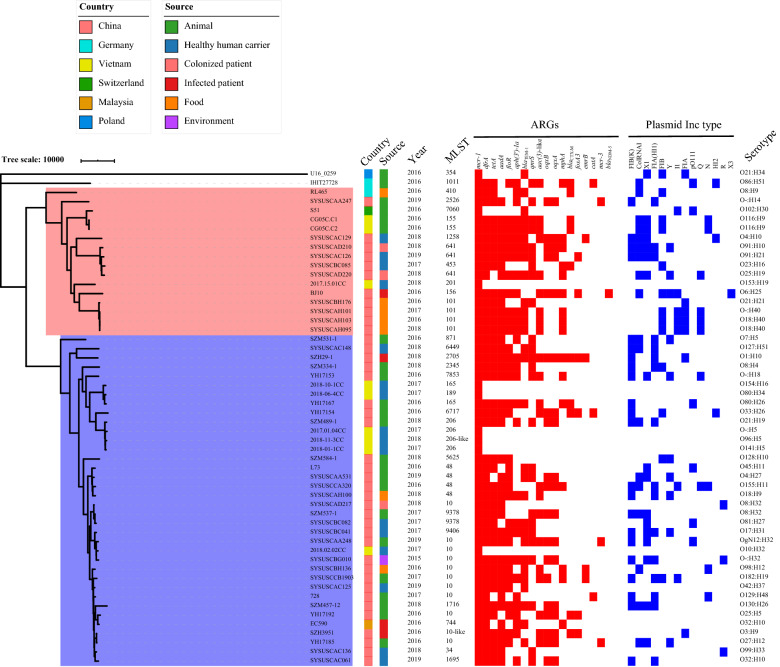
The phylogenetic tree and annotation of epidemiological and genomic features. The red colour range on the phylogenetic tree represents sequence cluster 1 (SC1), and the blue colour range represents SC2. The heatmap is showing the presence/absence of characters for antimicrobial resistance genes (ARGs) and plasmid Inc types

To systematically illustrate the genomic basis of chromosomally-encoded *mcr*-*1*-positive *E. coli* population, we collected other 30 *E. coli* genomes with chromosomally-encoded *mcr*-*1* from published literature for subsequent analysis (Additional file 1: Table S1). Through in silico multilocus sequence typing (MLST) assignment, 32 different sequence types (STs) within 10 ST complexes were determined (Fig. 1). The most common ST among chromosomally-encoded *mcr*-*1*-positive *E. coli* isolates was ST10 (n = 10, 18.5%), which is consistent with the main host for plasmid-mediated *mcr*-*1* on *E. coli* species [3, 4, 12]. The phylogeny demonstrated two sequence clusters (SCs), except for two isolates which were distinct from two SCs as the outgroup (Fig. 1). The sources and serotypes of these genomes were scattered on the phylogeny, suggesting that the emergence of chromosomally-encoded *mcr*-*1* was random without source- or lineage-based specificity (Fig. 1). Since most of the chromosomally-encoded *mcr*-*1*-positive *E. coli* isolates have been identified in China (n = 40, 74.1%), which was attributed to the extensive screening of *mcr*-*1* in China, the associations between locations and SCs was ambiguous (SC1 [11/16] vs SC2 [29/36], Fisher’s exact test, p = 0.49).

The *mcr*-*1* gene was initially found on plasmids in *Enterobacteriaceae* and on a transposon Tn*6330*, prompting that the chromosomally-encoded *mcr*-*1* could come from recombination of plasmid segments or transposition of Tn*6330* [13–15]. Therefore, we investigated the plasmidome of 54 genomes to illustrate the potential origin of chromosomally-encoded *mcr*-*1*. We identified 33 plasmid Inc types among all isolates, and the results showed that the most common Inc type was IncFIB(K) (45.8%, n = 22), followed by IncColRNAI (43.8%, n = 21), IncHI1 (33.3%, n = 16), IncX1 (31.3%, n = 15), IncFIB (AP001918) (27.1%, n = 13), and IncY (20.8%, n = 10). Remarkably, the common Inc types of *mcr*-*1*-harboring plasmids, such as IncX4, IncI2, IncHI2, and IncpO111 [1, 3, 4, 12], were rarely detected among these isolates (Fig. 1), indicating that the chromosomally-encoded *mcr*-*1* may derive from IS*Apl1*/Tn*6330* through transposition, but not from the plasmid.

We subsequently analyzed the genetic context of *mcr*-*1* for each isolate to investigate the genetic model of chromosomally-encoded *mcr*-*1*, except seven isolates were excluded due to short *mcr*-*1*-harboring contigs. We found that most of the *mcr*-*1* genes (93.6%, 44/47) were flanked by IS*Apl1*, comprising 24 isolates harboring upstream IS*Apl1* and 20 isolates carrying composite Tn*6330*, which complied with the hypothesis of transposition-mediated chromosome insertion.

**Fig. 2:**
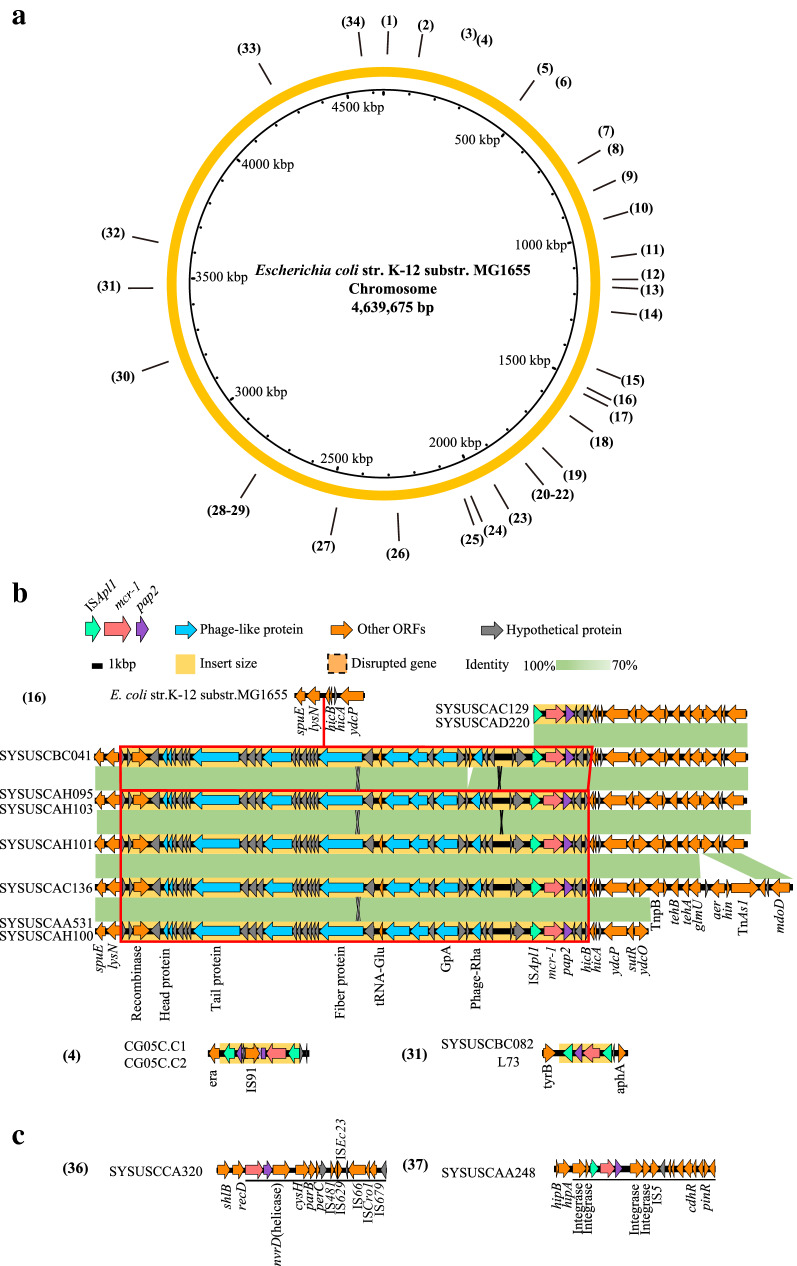
The insertion site and genomic patterns of chromosomally-encoded *mcr*-*1*. **a** The insertion patterns mapped to the *Escherichia coli* str. K-12 substr. MG1655 (Accession: NC_000913.2). The ring colored with orange represents the genome sequence of *Escherichia coli* str. K-12 substr. MG1655. The number in the outmost represents the order for each pattern, which showed in **b**, **c** and Additional file 2: Figure S1. **b** The genetic structure of chromosomally-encoded *mcr*-*1* patterns which included more than one isolate. **c** The genetic structure of chromosomally-encoded *mcr*-*1* which located on an integrative element region and a plasmid-like region

By mapping the insertion site onto the chromosome of *E. coli* MG1655, we noted that the distribution of chromosomally-encoded *mcr*-*1* insertion sites was sporadic (Fig. 2a). Thirty-seven clusters of *mcr*-*1*-harboring segments were generated based on sequence clustering analysis (Fig. 2a), which included three clusters involving more than one isolates (Fig. 2b) and 34 clusters only containing a single isolate (Additional file 2: Figure S1). The most common genetic pattern of chromosomally-encoded *mcr*-*1* (19.1%, 9/47) involves in an insertion segment in size of ~ 25.7 kb, containing an incomplete phage-like region (score = 40 for phage Vibrio 12B8 [NC_021073] by PHASTER) and a truncated Tn*6330* (IS*Apl1*-*mcr*-*1*-*pap2*), which was inserted into the *E. coli* genome between *lysN* and *hicB* (toxin-antitoxin system) loci (Fig. 2b). The incomplete phage-like region only contains head, tail, and fiber protein, and lacks some necessary functional components (Fig. 2b), which seems unfunctional under current conditions. We used BLASTn to search this phage-like sequence in NCBI non-redundant nucleotide database, and the results showed that only five sequences, which are located on *E. coli* chromosome, were identified with ≥ 60% coverage and ≥ 90% identity, indicating the correlation between chromosomally-encoded *mcr*-*1* and such phage-like region. Collectively, we heuristically concluded that such a phage-like region could mediate the emergence of chromosomally-encoded *mcr*-*1*, and then the phage may lose the lysogenic components, stabilization the genetic inheritance of chromosomally-encoded *mcr*-*1*. Additionally, the *mcr*-*1* of two isolates showed the insertion of *mcr*-*1* located on an integrative element region and a plasmid segment respectively, suggesting that chromosomally-encoded *mcr*-*1* could be derived from the integration of the integrative region and plasmid segment (Fig. 2c).

In conclusion, our study comprehensively investigated the genetic basis of chromosomally-encoded *mcr*-*1* in prevalence and potential mechanisms of lineage, plasmid, insertion sequence, and phage. Our results showed that chromosomally-encoded *mcr*-*1* was mainly derived from IS*Apl1* insertion in genomic locations sporadically. Notably, we reported a new transmission mechanism, a phage-like region without functional components, could associate with the emergence and stabilization of chromosomally-encoded *mcr*-*1*. The chromosomally-encoded *mcr*-*1* in current situations seems not a severe threat for public health, however, it could be a heritable repository and thrive again if the new selective pressure emerges, because the chromosome-mediated antimicrobial resistance genes (ARGs) might be conferred with genetic sustainability. In-depth investigations are needed to illustrate the genomic and epidemiological dynamics of chromosomally-encoded *mcr*-*1*, which may be changed after the approval of colistin in human clinical therapeutics in China [16].

## Literature searching

We searched PubMed using the terms of “*mcr*-*1*” [MeSH]/[All Fields] AND “chromosome” [MeSH]/[All Fields] AND “*Escherichia coli*” [MeSH]/[All Fields] for articles published before 1^th^ October 2020, and identified 20 publications, including 30 available *E. coli* genomes with chromosome-mediated *mcr*-*1* (Additional file 3: Figure S2).

### Bioinformatic analysis

Antimicrobial resistance genes screening, plasmid incompatibility typing and serotype identification were performed by Center for Genomic Epidemiology (http://www.genomicepidemiology.org/). Multilocus sequence typing (MLST) was assigned using Enterobase (http://enterobase.warwick.ac.uk/). Prophage prediction was implemented by PHASTER [17]. The phylogeny was constructed using RAxML v8.2 with GTR+G model and 1000 bootstrap [18] based on core genome single-nucleotide polymorphisms (cgSNPs) produced by Roary v3.11.2 and snp-site v2.4.1 [19]. Population structure was assessed using cgSNPs with hierBAPS [20]. The chromosome map was drawn by BRIG v0.95 and marked with insertion pattern manually by Easyfig v2.2.2 [21, 22]. The sequence clustering was performed by CD-HIT-EST [23].

### Statistical analysis

The significance of prevalence variation of chromosomally-encoded *mcr*-*1* between niches and epochs were tested by Fisher’s exact test using Statistical Package for the Social Sciences (SPSS), version 20.0.

## Supplementary information

**Additional file 1: Table S1.**

**Additional file 2: Figure S1.** The genetic structure of chromosomally-encoded *mcr-1* patterns which included only one isolate. The number for each pattern was identical to Fig. 2a.

**Additional file 3: Figure S2.** Flow diagram of the study selection process.

**Additional file 4: Appendix.**

## Data Availability

The datasets generated and analysed during the current study are available in the NCBI GenBank repository. The accession number for each genome can be obtained in Additional file 4: Appendix material.

## Abbreviations

*mcr*-*1*: Mobile colistin resistance gene
MLST: Multilocus sequence typing
STs: Sequence types
SCs: Sequence clusters
cgSNPs: Core genome single-nucleotide polymorphisms
ARG: Antimicrobial resistance gene
Inc: Incompatibility
