# Supplementary material for: Genomic patterns and characterizations of chromosomally-encoded mcr-1 in Escherichia coli populations
Source: Gut Pathog. 2020 Nov 28;12:55. doi: 10.1186/s13099-020-00393-2 (PMC7700713; doi:10.1186/s13099-020-00393-2)
Supplement: Supplementary file 1 — Additional file 1: Table S1. [file 13099_2020_393_MOESM1_ESM.docx]

| **Strain** | **Year** | **Country** | **Origin** | **Species** | **Sequencing strategy** | **Chromosome Accession** | **Plasmid Accession** | **Reference** |
| --- | --- | --- | --- | --- | --- | --- | --- | --- |
| YH17185 | 2018 | China | Chicken faeces | *Escherichia coli* | Illumina PE400 | GCF_002941765.1 | / | Within-host heterogeneity and flexibility of mcr-1 transmission in chicken gut |
| YH17167 | 2018 | China | Chicken faeces | *Escherichia coli* | Illumina PE400 | GCF_002941405.1 | / | Within-host heterogeneity and flexibility of mcr-1 transmission in chicken gut |
| YH17153 | 2018 | China | Chicken faeces | *Escherichia coli* | Illumina PE400 | GCF_002941545.1 | / | Within-host heterogeneity and flexibility of mcr-1 transmission in chicken gut |
| YH17154 | 2018 | China | Chicken faeces | *Escherichia coli* | Illumina PE400 | GCF_002941905.1 | / | Within-host heterogeneity and flexibility of mcr-1 transmission in chicken gut |
| YH17192 | 2018 | China | Chicken faeces | *Escherichia coli* | Illumina PE400 | GCF_002941065.1 | / | Within-host heterogeneity and flexibility of mcr-1 transmission in chicken gut |
| L73 | 2017 | China | Goose | *Escherichia coli* | PacBio | CP033378 | CP033379, CP033380 | Chromosome-mediated mcr-1 in Escherichia coli strain L73 from a goose |
| SZM531-1 | 2016 | China | Razor clam | *Escherichia coli* | Illumina PE150 | GCA_002270115.1 | / | Genetic basis of chromosomally-encoded mcr-1 gene |
| SZH3951 | 2016 | China | Human | *Escherichia coli* | PacBio | GCA_002270225.1 | / | Genetic basis of chromosomally-encoded mcr-1 gene |
| SZM537-1 | 2016 | China | Pork | *Escherichia coli* | Illumina PE150 | GCA_002270165.1 | / | Genetic basis of chromosomally-encoded mcr-1 gene |
| SZM489-1 | 2016 | China | Fish | *Escherichia coli* | Illumina PE150 | GCA_002270155.1 | / | Genetic basis of chromosomally-encoded mcr-1 gene |
| SZH29-1 | 2016 | China | Human | *Escherichia coli* | Illumina PE150 | GCA_002270125.1 | / | Genetic basis of chromosomally-encoded mcr-1 gene |
| SZM457-1 | 2016 | China | Pork | *Escherichia coli* | Illumina PE150 | GCA_002270245.1 | / | Genetic basis of chromosomally-encoded mcr-1 gene |
| SZM334-1 | 2016 | China | Pork | *Escherichia coli* | Illumina PE150 | GCA_002270255.1 | / | Genetic basis of chromosomally-encoded mcr-1 gene |
| SZM584-1 | 2016 | China | Pork | *Escherichia coli* | Illumina PE150 | GCA_002270175.1 | / | Genetic basis of chromosomally-encoded mcr-1 gene |
| 728 | 2017 | China | Chicken faeces | *Escherichia coli* | Illumina PE150 | MOKW00000000.1 | / | Comprehensive resistome analysis reveals the prevalence of NDM and MCR-1 in Chinese poultry production |
| RL465 | 2016 | Germany | Chicken meat | *Escherichia coli* | Illumina PE150 | FLKU01000080.1 | / | Chromosomal Locations of mcr-1 and blaCTX-M-15 in Fluoroquinolone-Resistant Escherichia coli ST410. |
| CG05C.C1 | 2017 | Vietnam | Chicken faeces | *Escherichia coli* | Illumina PE150 | FLZM00000000.1 (GCA_900094015.1) | / | Zoonotic Transmission of mcr-1 Colistin Resistance Gene from Small-Scale Poultry Farms, Vietnam. |
| IHIT27728 | 2017 | Germany | Reptile | *Escherichia coli* | Illumina PE150 | MACN00000000.1 | / | Imported reptiles as a risk factor for the global distribution of Escherichia coli harbouring the colistin resistance gene mcr-1. |
| CG05C.C2 | 2017 | Vietnam | Chicken faeces | *Escherichia coli* | Illumina PE150 | FLZI00000000.1 | / | Zoonotic Transmission of mcr-1 Colistin Resistance Gene from Small-Scale Poultry Farms, Vietnam. |
| S51 | 2015 | Switzerland(improted from Germany) | Raw chicken meat | *Escherichia coli* | PacBio | GCA_001660565.1 | / | Draft Genome Sequence of Escherichia coli S51, a Chicken Isolate Harboring a Chromosomally Encoded mcr-1 Gene. |
| BJ10 | 2016 | China | Human liver ascites | *Escherichia coli* | Illumina PE150 | LWQZ00000000.1 | / | Detection of mcr-1 colistin resistance gene in carbapenem-resistant Enterobacteriaceae (CRE) from different hospitals in China. |
| EC590 | 2016 | Malaysia | Human urine | *Escherichia coli* | PacBio | CP016182.2 | / | Emergence of mcr-1-mediated colistin resistance in Escherichia coli in Malaysia. |
| U16_0259 | 2016 | Poland | Pig faeces | *Escherichia coli* | Miseq | ERX2277398 (SRA) ERS2055691 | / | Occurrence and Characterization of mcr-1-Positive Escherichia coli Isolated From Food-Producing Animals in Poland, 2011–2016. |
| 2017.01.04CC | 2017 | Vietnam | Healthy human carrier | *Escherichia coli* | Nanopore | AP021891 | / | High Prevalence of Colistin-Resistant Escherichia coli with Chromosomally Carried mcr-1 in Healthy Residents in Vietnam. |
| 2018-01-1CC | 2018 | Vietnam | Healthy human carrier | *Escherichia coli* | Nanopore | AP021892 | / | High Prevalence of Colistin-Resistant Escherichia coli with Chromosomally Carried mcr-1 in Healthy Residents in Vietnam. |
| 2018.02.02CC | 2018 | Vietnam | Healthy human carrier | *Escherichia coli* | Nanopore | AP021896 | AP021897 | High Prevalence of Colistin-Resistant Escherichia coli with Chromosomally Carried mcr-1 in Healthy Residents in Vietnam. |
| 2018-06-4CC | 2018 | Vietnam | Healthy human carrier | *Escherichia coli* | Nanopore | AP021897 | / | High Prevalence of Colistin-Resistant Escherichia coli with Chromosomally Carried mcr-1 in Healthy Residents in Vietnam. |
| 2018-10-1CC | 2018 | Vietnam | Healthy human carrier | *Escherichia coli* | Nanopore | AP021894 | / | High Prevalence of Colistin-Resistant Escherichia coli with Chromosomally Carried mcr-1 in Healthy Residents in Vietnam. |
| 2018-11-3CC | 2018 | Vietnam | Healthy human carrier | *Escherichia coli* | Nanopore | AP021895 | / | High Prevalence of Colistin-Resistant Escherichia coli with Chromosomally Carried mcr-1 in Healthy Residents in Vietnam. |
| 2017.15.01CC | 2017 | Vietnam | Healthy human carrier | *Escherichia coli* | Nanopore | AP021890 | / | High Prevalence of Colistin-Resistant Escherichia coli with Chromosomally Carried mcr-1 in Healthy Residents in Vietnam. |
| SYSUSCAA247 | 2016 | China | Pig faeces | *Escherichia coli* | Illumina PE150 | SAMN14968775 | / | Dynamics of mcr-1 prevalence and mcr-1-positive Escherichia coli after the cessation of colistin use as a feed additive for animals in China: a prospective cross-sectional and whole genome sequencing-based molecular epidemiological study |
| SYSUSCAA248 | 2016 | China | Pig faeces | *Escherichia coli* | Illumina PE150 | SAMN15150488 | / | Dynamics of mcr-1 prevalence and mcr-1-positive Escherichia coli after the cessation of colistin use as a feed additive for animals in China: a prospective cross-sectional and whole genome sequencing-based molecular epidemiological study |
| SYSUSCAA531 | 2016 | China | Pig faeces | *Escherichia coli* | Illumina PE150 | SAMN15150489 | / | Dynamics of mcr-1 prevalence and mcr-1-positive Escherichia coli after the cessation of colistin use as a feed additive for animals in China: a prospective cross-sectional and whole genome sequencing-based molecular epidemiological study |
| SYSUSCAC061 | 2016 | China | Healthy human carrier | *Escherichia coli* | Illumina PE150 | SAMN15150490 | / | Dynamics of mcr-1 prevalence and mcr-1-positive Escherichia coli after the cessation of colistin use as a feed additive for animals in China: a prospective cross-sectional and whole genome sequencing-based molecular epidemiological study |
| SYSUSCAC125 | 2016 | China | Healthy human carrier | *Escherichia coli* | Illumina PE150 | SAMN15150491 | / | Dynamics of mcr-1 prevalence and mcr-1-positive Escherichia coli after the cessation of colistin use as a feed additive for animals in China: a prospective cross-sectional and whole genome sequencing-based molecular epidemiological study |
| SYSUSCAC126 | 2016 | China | Healthy human carrier | *Escherichia coli* | Illumina PE150 | SAMN15150492 | / | Dynamics of mcr-1 prevalence and mcr-1-positive Escherichia coli after the cessation of colistin use as a feed additive for animals in China: a prospective cross-sectional and whole genome sequencing-based molecular epidemiological study |
| SYSUSCAC129 | 2016 | China | Healthy human carrier | *Escherichia coli* | Illumina PE150 | SAMN15150493 | / | Dynamics of mcr-1 prevalence and mcr-1-positive Escherichia coli after the cessation of colistin use as a feed additive for animals in China: a prospective cross-sectional and whole genome sequencing-based molecular epidemiological study |
| SYSUSCAC136 | 2016 | China | Healthy human carrier | *Escherichia coli* | Illumina PE150 | SAMN15150494 | / | Dynamics of mcr-1 prevalence and mcr-1-positive Escherichia coli after the cessation of colistin use as a feed additive for animals in China: a prospective cross-sectional and whole genome sequencing-based molecular epidemiological study |
| SYSUSCAC148 | 2016 | China | Healthy human carrier | *Escherichia coli* | Illumina PE150 | SAMN15150495 | / | Dynamics of mcr-1 prevalence and mcr-1-positive Escherichia coli after the cessation of colistin use as a feed additive for animals in China: a prospective cross-sectional and whole genome sequencing-based molecular epidemiological study |
| SYSUSCAD210 | 2016 | China | Colonized patient | *Escherichia coli* | Illumina PE150 | SAMN15150496 | / | Dynamics of mcr-1 prevalence and mcr-1-positive Escherichia coli after the cessation of colistin use as a feed additive for animals in China: a prospective cross-sectional and whole genome sequencing-based molecular epidemiological study |
| SYSUSCAD217 | 2016 | China | Colonized patient | *Escherichia coli* | Illumina PE150 | SAMN15150497 | / | Dynamics of mcr-1 prevalence and mcr-1-positive Escherichia coli after the cessation of colistin use as a feed additive for animals in China: a prospective cross-sectional and whole genome sequencing-based molecular epidemiological study |
| SYSUSCAD220 | 2016 | China | Colonized patient | *Escherichia coli* | Illumina PE150 | SAMN15150498 | / | Dynamics of mcr-1 prevalence and mcr-1-positive Escherichia coli after the cessation of colistin use as a feed additive for animals in China: a prospective cross-sectional and whole genome sequencing-based molecular epidemiological study |
| SYSUSCAH095 | 2016 | China | Food | *Escherichia coli* | Illumina PE150 | SAMN15150499 | / | Dynamics of mcr-1 prevalence and mcr-1-positive Escherichia coli after the cessation of colistin use as a feed additive for animals in China: a prospective cross-sectional and whole genome sequencing-based molecular epidemiological study |
| SYSUSCAH100 | 2016 | China | Food | *Escherichia coli* | Illumina PE150 | SAMN15150500 | / | Dynamics of mcr-1 prevalence and mcr-1-positive Escherichia coli after the cessation of colistin use as a feed additive for animals in China: a prospective cross-sectional and whole genome sequencing-based molecular epidemiological study |
| SYSUSCAH101 | 2016 | China | Food | *Escherichia coli* | Illumina PE150 | SAMN15150501 | / | Dynamics of mcr-1 prevalence and mcr-1-positive Escherichia coli after the cessation of colistin use as a feed additive for animals in China: a prospective cross-sectional and whole genome sequencing-based molecular epidemiological study |
| SYSUSCAH103 | 2016 | China | Food | *Escherichia coli* | Illumina PE150 | SAMN15150502 | / | Dynamics of mcr-1 prevalence and mcr-1-positive Escherichia coli after the cessation of colistin use as a feed additive for animals in China: a prospective cross-sectional and whole genome sequencing-based molecular epidemiological study |
| SYSUSCBC041 | 2017 | China | Healthy human carrier | *Escherichia coli* | Illumina PE150 | SAMN15150503 | / | Dynamics of mcr-1 prevalence and mcr-1-positive Escherichia coli after the cessation of colistin use as a feed additive for animals in China: a prospective cross-sectional and whole genome sequencing-based molecular epidemiological study |
| SYSUSCBC082 | 2017 | China | Healthy human carrier | *Escherichia coli* | Illumina PE150 | SAMN15150504 | / | Dynamics of mcr-1 prevalence and mcr-1-positive Escherichia coli after the cessation of colistin use as a feed additive for animals in China: a prospective cross-sectional and whole genome sequencing-based molecular epidemiological study |
| SYSUSCBC085 | 2017 | China | Healthy human carrier | *Escherichia coli* | Illumina PE150 | SAMN15150505 | / | Dynamics of mcr-1 prevalence and mcr-1-positive Escherichia coli after the cessation of colistin use as a feed additive for animals in China: a prospective cross-sectional and whole genome sequencing-based molecular epidemiological study |
| SYSUSCBG010 | 2017 | China | Environment | *Escherichia coli* | Illumina PE150 | SAMN15150506 | / | Dynamics of mcr-1 prevalence and mcr-1-positive Escherichia coli after the cessation of colistin use as a feed additive for animals in China: a prospective cross-sectional and whole genome sequencing-based molecular epidemiological study |
| SYSUSCBH136 | 2017 | China | Food | *Escherichia coli* | Illumina PE150 | SAMN15150507 | / | Dynamics of mcr-1 prevalence and mcr-1-positive Escherichia coli after the cessation of colistin use as a feed additive for animals in China: a prospective cross-sectional and whole genome sequencing-based molecular epidemiological study |
| SYSUSCBH176 | 2017 | China | Food | *Escherichia coli* | Illumina PE150 | SAMN15150508 | / | Dynamics of mcr-1 prevalence and mcr-1-positive Escherichia coli after the cessation of colistin use as a feed additive for animals in China: a prospective cross-sectional and whole genome sequencing-based molecular epidemiological study |
| SYSUSCCA320 | 2018 | China | Pig faeces | *Escherichia coli* | Illumina PE150 | SAMN15150509 | / | Dynamics of mcr-1 prevalence and mcr-1-positive Escherichia coli after the cessation of colistin use as a feed additive for animals in China: a prospective cross-sectional and whole genome sequencing-based molecular epidemiological study |
| SYSUSCCB1903 | 2018 | China | Pig faeces | *Escherichia coli* | Illumina PE150 | SAMN15150510 | / | Dynamics of mcr-1 prevalence and mcr-1-positive Escherichia coli after the cessation of colistin use as a feed additive for animals in China: a prospective cross-sectional and whole genome sequencing-based molecular epidemiological study |
