# Supplementary figures and images for: Genomic patterns and characterizations of chromosomally-encoded mcr-1 in Escherichia coli populations
Source: Gut Pathog. 2020 Nov 28;12:55. doi: 10.1186/s13099-020-00393-2 (PMC7700713; doi:10.1186/s13099-020-00393-2)

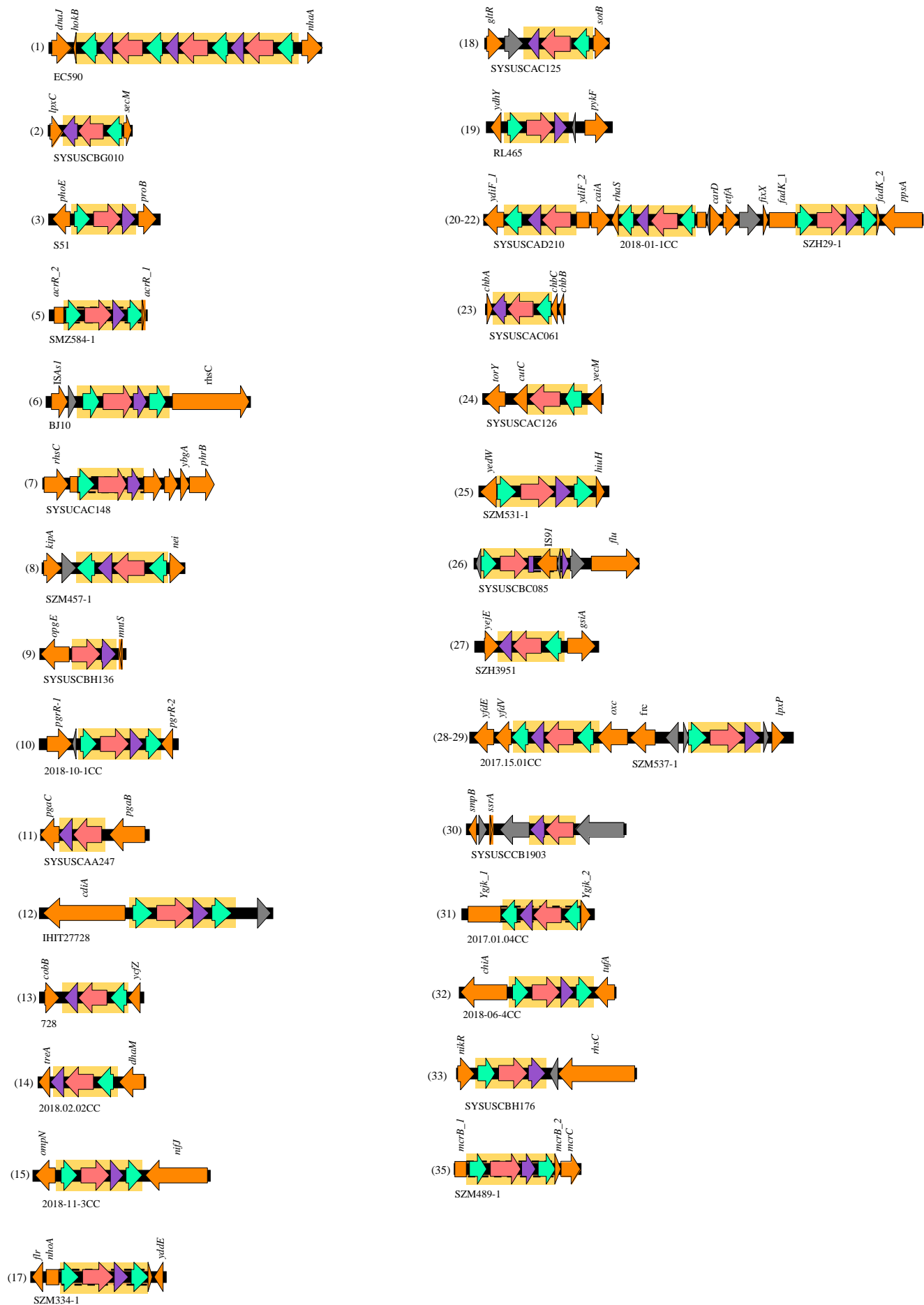

Figure S1. The genetic structure of chromosomal *mcr-1* patterns which included only one isolate.

Supplement: Supplementary file 2 — Additional file 2: Figure S1. The genetic structure of chromosomally-encoded mcr-1 patterns which included only one isolate. The number for each pattern was identical to Fig. 2a. [file 13099_2020_393_MOESM2_ESM.pdf]
