# Supplementary material for: Genomic patterns and characterizations of chromosomally-encoded mcr-1 in Escherichia coli populations
Source: Gut Pathog. 2020 Nov 28;12:55. doi: 10.1186/s13099-020-00393-2 (PMC7700713; doi:10.1186/s13099-020-00393-2)
Supplement: Supplementary file 3 — Additional file 3: Figure S2. Flow diagram of the study selection process. [file 13099_2020_393_MOESM3_ESM.pdf]

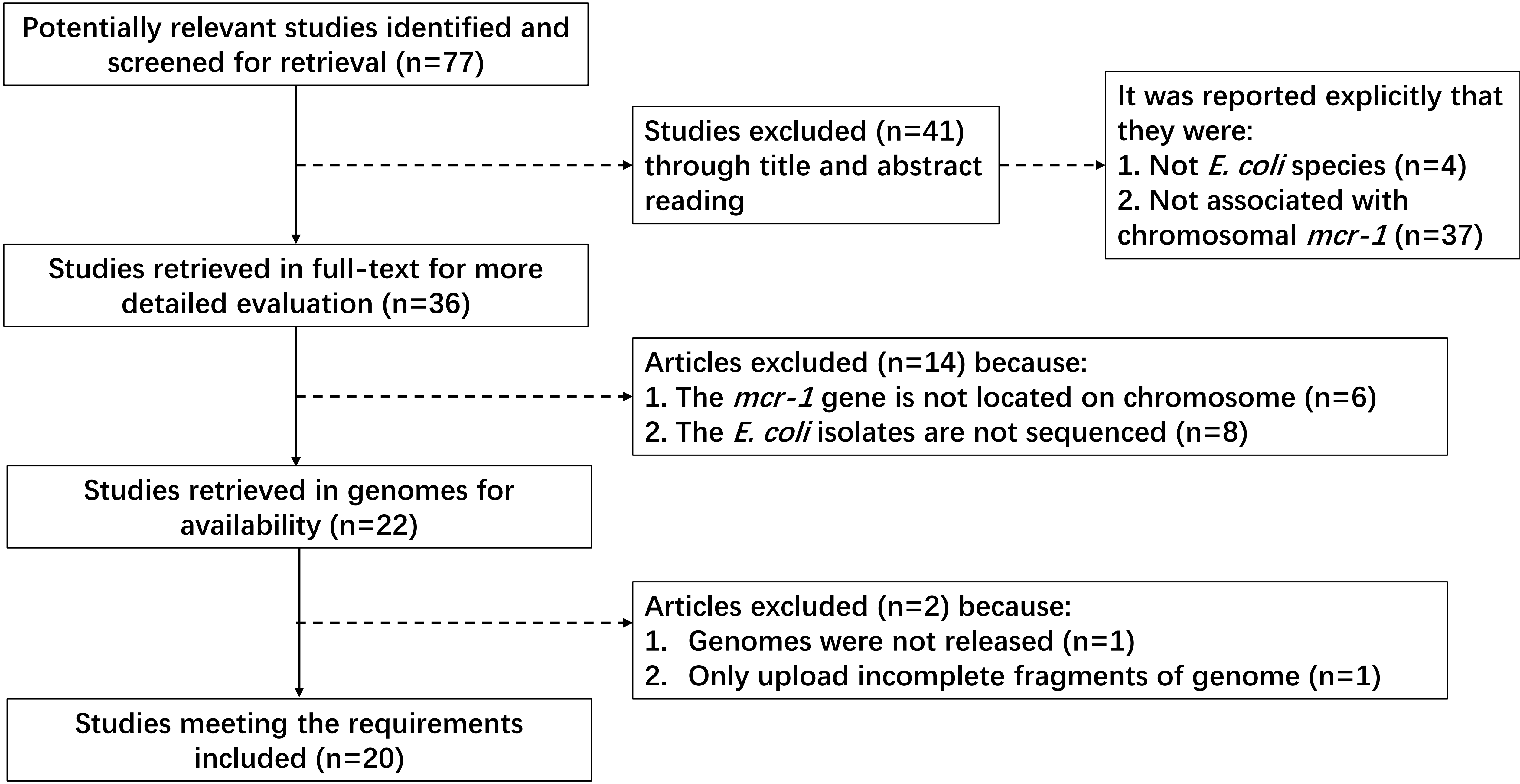
Potentially relevant studies identified and screened for retrieval (n=77)
Studies excluded (n=41) through title and abstract reading
It was reported explicitly that they were:
1. Not *E. coli* species (n=4)
2. Not associated with chromosomal *mcr-1* (n=37)
Studies retrieved in full-text for more detailed evaluation (n=36)
Articles excluded (n=14) because:
1. The *mcr-1* gene is not located on chromosome (n=6)
2. The *E. coli* isolates are not sequenced (n=8)
Studies retrieved in genomes for availability (n=22)
Articles excluded (n=2) because:
1. Genomes were not released (n=1)
2. Only upload incomplete fragments of genome (n=1)
Studies meeting the requirements included (n=20)
